# Digital integration between hospitals and local health authorities for enhanced vaccination coverage among frail patients: the CareVax study protocol

**DOI:** 10.3389/fpubh.2025.1490244

**Published:** 2025-01-30

**Authors:** Alberto Lontano, Luca Regazzi, Diego Maria Tona, Matteo Di Pumpo, Martina Porcelli, Maria Gabriella Cacciuttolo, Paolo Parente, Antonio Gasbarrini, Giuseppe Grandaliano, Nicola Panocchia, Loris Lopetuso, Tina Pasciuto, Chiara Cadeddu, Stefania Bruno, Patrizia Laurenti, Domenico Pascucci, Roberta Pastorino

**Affiliations:** ^1^Section of Hygiene, University Department of Health Sciences and Public Health, Faculty of Medicine, Università Cattolica del Sacro Cuore, Rome, Italy; ^2^Local Health Authority ASL Roma 1, Rome, Italy; ^3^Department of Translational Medicine and Surgery, Università Cattolica del Sacro Cuore, Rome, Italy; ^4^Department of Medical and Surgical Sciences, Fondazione Policlinico Universitario A. Gemelli IRCCS, Rome, Italy; ^5^Research Core Facility Data Collection G-STeP, Fondazione Policlinico Universitario A. Gemelli IRCCS, Rome, Italy; ^6^Erasmus School of Health Policy & Management, Erasmus University Rotterdam, Rotterdam, Netherlands; ^7^Department of Women, Child and Public Health, Fondazione Policlinico Universitario A. Gemelli IRCCS, Rome, Italy; ^8^Health Management, Fondazione Policlinico Universitario A. Gemelli IRCCS, Rome, Italy

**Keywords:** vaccination, digital public health, hospital-territory integration, fragility, primary prevention

## Abstract

**Background:**

The 2022–2025 Italian Plan for vaccine prevention (PNPV), recognizes vaccine-preventable diseases (VPDs) as significant contributors to mortality, morbidity, and healthcare expenditure. The digitalization of the national vaccine registry is underway. Initiatives aimed at enhancing digital integration between hospitals and territories are limited, and there is still a gap in the development of automated systems for identifying patients who could benefit from vaccinations directly offered from hospitals.

**Methods:**

Adult frail patients who access the hospital will be recruited over 4 years, following the acquisition of informed consent. With the assistance of a privacy-preserving automated algorithm, electronic hospital and vaccination records will be utilized to assess eligibility for vaccinations against *SARS-CoV-2*, *Herpes Zoster*, *Influenza*, *Streptococcus pneumoniae,* and *Hepatitis B*. Eligible patients will be invited to schedule a vaccination appointment and will be asked to fill in a questionnaire evaluating patient-reported experience measures (PREMs). Outcomes of interest are the feasibility of the pathway, patients’ satisfaction and concerns with it, and its impact on vaccination coverage.

**Ethics and dissemination:**

The study has been approved by the ethics committee of the “Fondazione Policlinico Universitario Agostino Gemelli” -FPG- (comitato.etico@policlinicogemelli.it), with approval number 5819. Furthermore, it has been published on ClinicalTrial.gov with the approval number NCT06127563. The results of the study will be disseminated via conference presentations and peer-reviewed publications.

**Clinical trial registration:**

ClinicalTrials.gov, identifier NCT06127563.

## 1 Introduction

The 2022–2025 Italian Plan for vaccine prevention (PNPV, Piano Nazionale Prevenzione Vaccinale), furthering the legacy of the 2017–2019 PNPV and accordingly with the guidelines of the 2030 Agenda for Immunization, recognizes vaccine-preventable diseases (VPDs) as significant contributors to mortality, morbidity and healthcare expenditure. It also defines populations at higher risk of developing VPDs, susceptible to complications and severe sequelae (1–3).

Individuals at increased risk include those with chronic diseases (e.g., cardiorespiratory, neurological, metabolic, neoplastic, and immunological diseases), severe disabilities (physical, sensory, intellectual, or mental), pregnant women, and those more susceptible to severe disease due to age (4).

The Global Vaccine Action Plan 2011–2020 urged countries to achieve a 90% coverage threshold with all vaccines in their national immunization program by 2020 (4). In this context the pandemic had mixed effects, accelerating the development of innovative prevention paradigms such as COVID-19 online vaccination booking and in-hospital immunization while, on the other hand, leading to a significant decline in vaccination coverage across all age groups (5).

In response, the PNPV underscores the urgency of implementing initiatives for accessible vaccination options, bringing them closer to patients at treatment centers, including Hospital, General Practitioners’ offices and Community Health Houses (6–8).

Since the reform of Title V of the Constitution in 2001, delocalizing competence over healthcare, there has been a marked regionalization of the Italian health system, with only a few regions being able to boast collaborative examples between local health authorities and hospitals to enhance vaccination coverage in particular categories of patients. Even then these models often lack the feasibility for broader application to other contexts and do not rely on integrated information systems to improve efficiency and optimize the allocation of resources (9).

Mission 6 of the National Recovery and Resilience Plan (PNRR) aims to capitalize on healthcare delivery opportunities presented by technological innovations, emphasizing the analysis of clinical and administrative data flows (10). Italy’s delayed digitization process in healthcare, compared to other European countries, necessitates a strategic focus on technology-enhanced healthcare initiatives (11).

Currently, Italy is in the process of digitizing the Vaccine Registry in accordance with the 2022–2025 PNPV, intending to create a Digital Vaccine Record. Accessibility varies across regions, with some offering comprehensive access through Electronic IDs (e.g., SPID, Public system for electronic identification) (12) while others limit access to anti-COVID-19 vaccinations (13, 14) or require contact with the Health District. Lombardy region (14) even provides vaccination information via a QR code, akin to the European Green Pass for anti-SARS-CoV-2 vaccination.

Regarding vaccine booking, some regions offer digital platforms (15–17), but no automated systems are in place to identify patients eligible for vaccination based on specific risk factors.

The challenge lies in improving the identification of patients eligible for vaccination, a key barrier to better vaccination strategies for frail populations. Currently, no fully automated digital systems to identify eligible patients within integrated frameworks are described in the literature. However, existing examples of hospital-based vaccination show promising potential and could serve as foundational steps toward increasingly digitalized models.

Hospital-based vaccination is critical for reaching at-risk groups, encouraging vaccine uptake through patient trust in healthcare providers, and benefiting from hospital logistics. In Italy, while initiatives such as those described by Veronese et al. (18) and Ridolfi et al. (19) show promise, they remain largely manual and lack integration with community health systems.

Globally, similar challenges exist. In the United States, Canada, and Australia, hospital-based programs for vaccines like influenza and pneumococcal disease are common but lack automation and integration with national registries (20). For instance, a 2023 Portuguese study (21) on diabetic patients used hospital reminders but required vaccines to be administered in community centers. Similarly, the PANDA II study (22) in China provided hospital-based influenza vaccinations but lacked automation or coordination with community services.

In the United States, interventions such as nurse-led models, patient reminder letters, and physician prompts have improved vaccination rates (23, 24). However, these efforts are not automated or integrated with local health systems, limiting their scalability and overall impact.

### 1.1 Primary objectives

The CareVax study (Caring for frail patients through vaccination) aims to evaluate the feasibility of implementing a privacy-preserving integrated hospital-territory pathway for vaccinations against influenza, pneumococcal disease, Herpes Zoster, Severe Acute Respiratory Syndrome Coronavirus 2 (SARS-CoV-2), and Hepatitis B Virus (HBV). These vaccinations have been selected through a joint evaluation with the involved hospital departments and the competent Local Health Authority (ASL Roma 1) because they are included in the national prevention plan, lack sufficient coverage at the local level, and can help reduce the burden of care for the participating hospital departments, thereby increasing safety, quality, and appropriateness of care. The underlying hypothesis is that the establishment of an in-hospital pathway, supported by an automated alert system for clinicians, may facilitate the development of a personalized vaccination program for vulnerable patients requiring hospital care.

### 1.2 Secondary objectives

The secondary objectives of this study are to verify the effectiveness of enhancing vaccination coverage among frail patients and to assess their opinions on vaccination and the vaccination pathway.

## 2 Methods

### 2.1 Study design

CareVax is designed as a Single-Arm, Pre-Post Intervention Study.

### 2.2 Population

The CareVax model will be tested within the teaching hospital Fondazione Policlinico Universitario Agostino Gemelli IRCCS (FPG) located in the Metropolitan Area of Rome (in the Lazio Region). The departments of Internal Medicine & Gastroenterology, Geriatric Internal Medicine, Nephrology, Gynecologic Oncology, and Dermatology have agreed to partake in the study, hence only their patients will be enrolled.

### 2.3 Study duration

The study will last for 4 years.

Patient recruitment will occur throughout the entire study period, utilizing a dynamic cohort approach.

### 2.4 Inclusion criteria

Patients who:

- are treated in one of the departments involved in the study.
- are over 18 years old.
- have their medical residence in Lazio Region, as only the regional vaccine registry is accessible.
- provide informed consent to be enrolled in the study.

### 2.5 Procedures and collected variables

**Figure 1:**
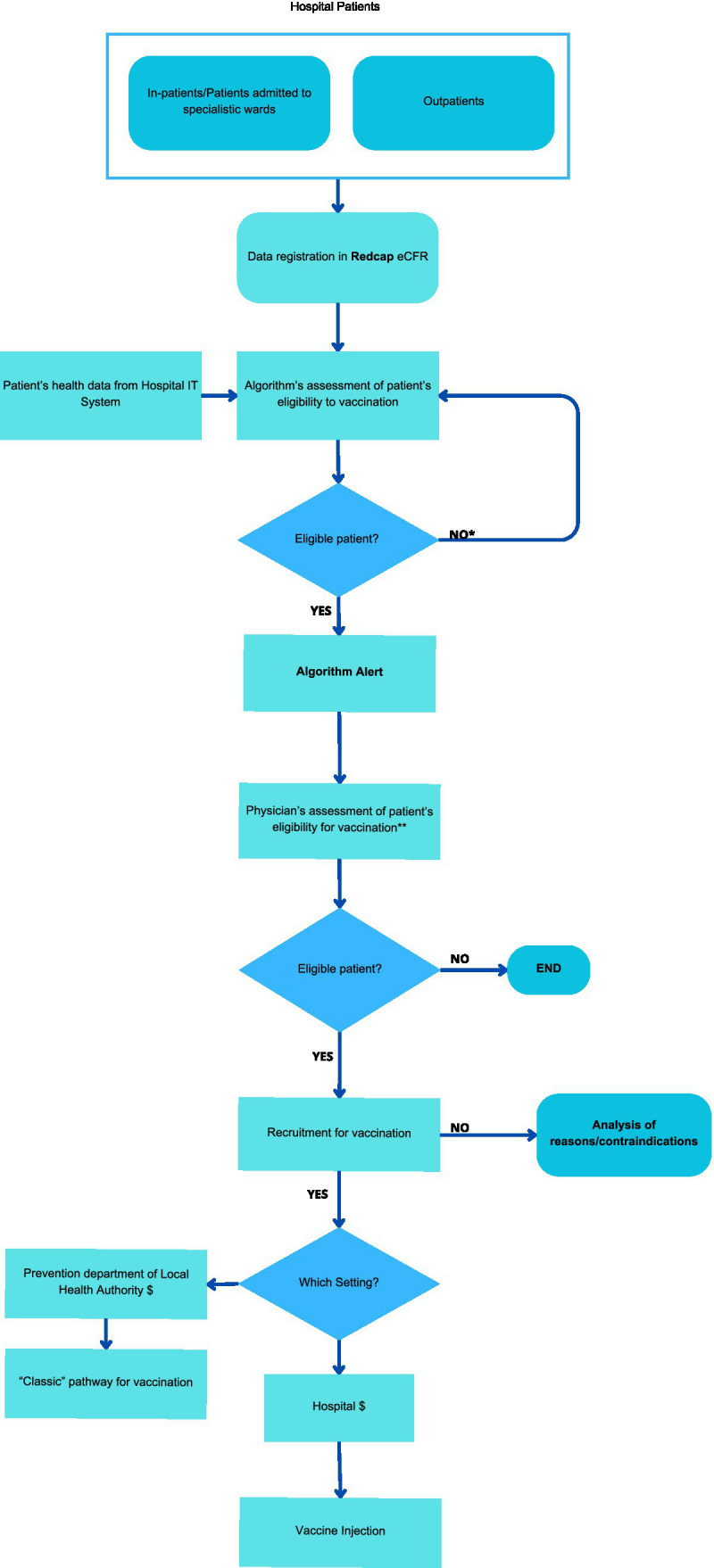
Personalized vaccine pathway.

The personalized vaccine pathway, exemplified in Figure 1, involves the following procedures and collected variables:

I Informed consent for algorithm screening and patient contact:

- Patients visiting the inpatient and outpatient clinics of the participating departments will be informed about the study. Those who express interest in participating will provide written informed consent, which will be recorded using a REDCap electronic Case Report Form (eCRF). This consent will enable the retrievement of information on their health status, relevant risk factors, and vaccination history from the Institutional ERP (Enterprise Resource Planning) TrackCare. The data will then be analyzed by a well defined algorithm to determine their eligibility for a personalized vaccine offer. Additional details about the use of the REDCap eCRF are provided in a dedicated section.
- The consent will also grant FPG personnel the permission to contact patients feligible for specific vaccinations according to the algorithm output.

II Algorithm operation:

- The algorithm, based on national guidelines on vaccinations, utilizes a decision tree incorporating data on age, season (e.g., for influenza), Regional Disease Exemption Codes (25) and ICD9-CM (26) codes for procedures and diseases increasing the risks posed by VPDs (such as cardiovascular diseases, diabetes, immunosuppressive drugs therapy, malignant neoplasms lung diseases, kidney failure or dialysis). The algorithm has already been developed using SAS code by the Information and Communication Technology team at FPG, accessing data directly from the hospital’s IT system. It operates in with a privacy preserving approach exclusively on patients who have provided informed consent.
- Data retrieved from the Hospital Information System are cross-referenced with the Regional Vaccine Registry to exclude vaccines already received.
- If direct information is unavailable (for example pregnancy status, type of diabetes), surrogate data, such as access to gynecology-obstetrics departments or recent delivery, may be used.

III Algorithm output:

- The algorithm will periodically generate a list of patients eligible for each vaccine, which will be recorded directly in the eCRF. This list will present their health records and highlight the specific conditions that warrant vaccination.
- Physicians at the FPG vaccination center will review this list, flagged by an alert on the eCRF, and, in accordance with the PNPV national guidelines (3), reassess vaccination indications to determine the appropriate administration setting.

IV Patient contact and vaccination sessions:

- Eligible patients will be contacted directly by physicians from the vaccination center via phone calls (mobile or landline) to assess their interest in receiving the recommended vaccinations. Patients will be able to discuss the benefits and risks of the proposed vaccination(s).
- Vaccination sessions will be booked by the physician who contacts the patient, based on the severity of the patient’s condition and the physician’s clinical judgment. Patients with more complex conditions will be scheduled for vaccination at the hospital vaccination center of FPG, while those with less severe conditions will be referred to vaccine centers of Local Health Authority ASL Roma 1. Based on the appointment date, patients will arrive to receive the vaccination in the setting determined at the time of booking.
- Where feasible, a co-administration schedule for multiple vaccines in the same session will be employed, as performed during previous vaccination campaigns at FPG (27).

V Clinical reassessment and informed consent:

- In both hospital and local health authority settings, vaccination physicians will reassess the vaccination indications for algorithm-identified patients.
- Vaccination physicians will administer informed consent specific to vaccinations.

VI Post-vaccination questionnaire:

- Following vaccination, patients will complete a questionnaire assessing satisfaction with organizational aspects, healthcare personnel, and opinions on vaccinations (Supplementary File S1) using the Health Belief Model domains (28), which will also be recorded in the eCRF.

A pilot study will be conducted with 10 participants to ensure that all aspects of the personalized vaccine pathway are thoroughly evaluated and validated. This initial phase aims to identify potential challenges and refine the procedures, thereby enhancing the overall feasibility of the pathway prior to its full implementation.

### 2.6 Patient protection and confidentiality

The processing of the personal data of patients taking part in the study, and, in particular, regarding data concerning consent, shall comply with European Regulation on the Privacy of data (UE 2016/679) (29). Investigators will guarantee that all persons involved in this study will respect the confidentiality of any information concerning the study. All parties involved in this study will maintain the strict confidentiality to assure that neither the person nor the family privacy of the patient participating in the study is violated; appropriate measures shall be taken to avoid the access of non-authorized persons to the study data. The patient can withdraw consent whenever s/he wants and further data will not be collected (unless they accept to still be contacted for long term outcomes), even if the already collected data will be used for the analyses of the study. Clinical data already collected will be destroyed only if the right to be forgotten will be requested according to GDPR-22 (30). In this case, to comply with regulatory obligations, the progressive patient ID, will be maintained, and the reason for withdrawal will be recorded in the electronic eCRF.

### 2.7 Data collection and management

A customized eCRF will be created for the study. Data will be collected and managed using REDCap electronic data capture tools hosted at https://redcap-irccs.policlinicogemelli.it/. The REDCap (Research Electronic Data Capture) is a secure, web-based software platform fully compliant with the 21 CFR Part 11 and GDPR and designed to support data capture for research studies, providing:

- an intuitive interface to acquire validated data;
- audit trail to monitor data handling and export procedures;
- automated export procedures to download data into common statistical software;
- data integration procedures and interoperability with external sources (31).

The research core facility Data Collection of the Scientific Technology Park of Fondazione Policlinico Universitario A. Gemelli-IRCCS is in charge for the management of REDCap platform used for the study. This facility is focused on collecting and managing research data for all no-profit projects of the above mentioned Institution in compliance with good clinical practices (GCP), current legislation on data protection (GDPR), and data quality, assessed through Accuracy, Completeness, Consistency, Integrity, and Timing (ACCIT) criteria (32–34).

Recorded information is confidential and the database is privacy-protected; i.e., no data can be traced back to the patient in research reports and no unauthorized individuals may have access to the data about individuals in the database. Only people officially registered as investigators or data managers will receive a user login to access with a multifactorial authentication the REDCap web platform and enter/manage data.

### 2.8 Data quality and standards

The eCRF will be designed according to protocol, dataset set-up and validation, edit checks programming. Only when reviewed and fully tested, the dataset will be activated to receive the data. The Investigators involved in the study will be responsible to ensure that the CRFs are properly and completely filled in. CRFs must be completed for all patients who have given informed consent. Sources of clinical information include the physician’s patient record, hospital notes, original laboratory records. Data will be entered into the CRF in a truthful, accurate and timely manner and each participating Investigator will be responsible for ensuring data quality. Personal medical information may be reviewed to ensure patients’ safety and will always be treated as confidential. Whenever possible, data will be imported directly from institutional management systems through interoperability processes.

During data collection a remote monitoring and data quality rules will be activated to manage discrepancies and inconsistencies, and to generate queries. The “Data Resolution Workflow” module will allow a workflow for documenting the process of resolving issues with data in the project (i.e., opening, responding to, and closing data queries). Different user privileges may be given to users that control whether users can view, open/close, or respond to data queries.

### 2.9 Endpoints

The feasibility of the pathway will be assessed on various levels, encompassing the integration of digital systems, the practicality of proposed interventions to implement the integrated hospital-territory pathway, patient participation, and the effectiveness of enhancing vaccination coverage.

#### 2.9.1 Primary endpoints

##### 2.9.1.1 Integration of digital system

- Concordance between the clinician’s and the algorithm’s judgments on patient eligibility for vaccination, measured on a sample of patients.
- Proportion of vaccinations confirmed by physicians compared to those proposed by the algorithm.

##### 2.9.1.2 Practicality of proposed interventions

- Number of patients successfully contacted to offer a vaccination appointment after being considered eligible compared to the number of patients flagged by the algorithm.

##### 2.9.1.3 Patient participation

- Number of patients consenting to vaccination appointments compared to the number of patients contacted.
- Number of patients presenting to vaccination centers (Hospital or Local Health Authority) compared to the number who have booked a vaccination appointment.
- Number of patients who drop out or are lost to follow-up versus the total number of patients recruited in the study.
- Number of patients who agree or strongly agree with the questionnaire’s statements on the algorithm, the clinical pathway, and the medical personnel (Sections A and B of the attached questionnaire – Supplementary File S1).

#### 2.9.2 Secondary endpoints

- N° anti-influenza vaccinations at 6, 12, 24 months.
- N° anti-pneumococcal vaccinations at 6, 12, 24 months.
- N° anti-Herpes Zoster vaccinations at 6, 12, 24 months.
- N° anti-SARS-CoV-2 vaccinations at 6, 12, 24 months.
- N° anti-HBV vaccinations at 6, 12, 24 months.
- Percentage increase in the number of patients vaccinated against influenza/Pneumococcus/Herpes Zoster/SARS-CoV-2/HBV at 6, 12, and 24 months compared to the baseline vaccination rate, measured within the same cohort of patients. This baseline will be established from the initial vaccination rates within the same cohort of patients enrolled prior to the implementation of the personalized vaccine pathway.

### 2.10 Statistical plan

#### 2.10.1 Sample size calculation

Participation in the study will be offered to all eligible patients referred to the involved departments during the study period, with an expected number of 1,500 subjects. This sample size is sufficient to detect a 10% increase in vaccination coverage from 50% before the intervention to 60% after the intervention, with a significance level of 0.05 and a power of 80% using McNemar’s test for paired data.

#### 2.10.2 Statistical analysis

The sample recruited in the study will be described in its clinical and demographic characteristics using descriptive statistical techniques. In particular, qualitative data will be expressed as absolute and relative percentage frequency, while quantitative variables with mean and standard deviation or median and interquartile range, as appropriate. Algorithm-physician agreement will be evaluated with Cohen’s k coefficient. To evaluate changes in vaccination coverage before and after the implementation of the new pathway, we will use McNemar’s test for paired data. For each vaccination the comparison with baseline values will be conducted at 6, 12 and 24 months post-implementation. Additionally, to evaluate the impact of different factors on vaccination uptake over time, we will use a mixed-effects logistic regression model. This model will account for both fixed effects (such as time, gender, and age) and random effects (to handle the correlation of repeated measures within participants). We will also consider additional potential confounding factors such as socioeconomic status, access to healthcare, and prior vaccination history. By including these variables, we will examine whether they contribute to variations in vaccination coverage over time, identify significant trends or disparities, and adjust for any confounding influences that might affect the outcomes of the vaccination program.

Descriptive statistics will be performed on the questionnaire designed to assess satisfaction with organizational aspects, healthcare personnel, and opinions on vaccinations (Supplementary File S1).

The statistical analysis will be conducted using StataCorp 2023. Stata Statistical Software: Release 18. College Station, TX: StataCorp LLC.

## 3 Discussion

The CareVax study endeavors to assess the feasibility of establishing an integrated hospital-territory pathway for vaccinations targeting influenza, pneumococcal disease, Herpes Zoster, Severe Acute Respiratory Syndrome Coronavirus (SARS-CoV-2), and Hepatitis B Virus (HBV) for vulnerable patient populations, intercepted in hospital and supported by the assistance of an automated alert system.

As the place where a significant portion of frail patients seek treatment, the hospital setting may represent a potential focal point for improving vaccine coverage. However, even if about 50% of hospital patients have been estimated as being eligible for pneumococcal vaccination, as of today limited efforts have been directed toward reaching this population (35). The provisions of the new PNPV seek to address this gap by promoting a vaccine distribution strategy aligned with patients’ points of care.

Hospitals, often overlooked as vaccination sites in Italy, can play a pivotal role in this process. Various hospital-based vaccination plans have already proven effective, including opportunistic vaccination pathways and the utilization of standard operating procedures to enable allied professionals to gather patient history, obtain consent, and administer vaccines themselves (36–38). Similarly, automated algorithms have been validated to identify eligible patients for vaccination, presenting a promising tool to remind clinicians of the possibility of offering specific vaccinations to selected patients (24, 39, 40).

CareVax has macro-level implications, as it could represent a exemplary organizational model, useful for cost containment and computerized exportability in various public health domains beyond vaccinations. At the meso level, it constitutes a fundamental tool for increasing vaccination coverage and achieving vaccination coverage goals set by Regions and, consequently, Local Health Authorities within the Vaccination Prevention Plans. A similar integrated model might serve as a stimulus for mapping the information systems of hospital and territorial facilities, identifying common elements and obstacles to be resolved to ensure their interoperability.

Finally, at the micro level, CareVax represents an opportunity to improve the quality of care and patient satisfaction, increasing their engagement and participation in primary prevention programs. Similarly, such a project increases awareness and enables the active involvement of clinicians in primary prevention practices, which are often overlooked in favor of less effective secondary or tertiary prevention practices.

CareVax effectively addresses several shortcomings in existing international vaccination protocols by implementing a fully integrated digital infrastructure that seamlessly connects hospital and community health services. Unlike the manual or partially digitized systems commonly observed in other countries (20–24), CareVax leverages an advanced automated algorithm to identify at-risk patients based on their clinical profiles and vaccination histories, minimizing delays and inefficiencies. By streamlining patient selection and enabling the delivery of personalized vaccination offers, this system empowers dedicated clinicians to efficiently manage alerts, follow up with patients, and provide tailored vaccination options directly within the hospital setting. This integrated and innovative approach not only enhances accessibility and adherence but also lays the groundwork for broader public health improvements through better vaccination outcomes.

This protocol builds upon this foundation by presenting the design of a comprehensive computerized system aimed at increasing vaccination coverage, a step forward compared to the analogous or non-integrated experiences previously described in the literature (18–24). In addition, our algorithm holds promise for cost-effective implementation in major Italian hospitals and might serve as a potential model for smaller institutions. Furthermore, other strengths of our study lie in the extensive patient volume treated in the FPG, which can provide a robust study sample, and the extended duration of the study, which enables the assessment of the program’s impact over time.

However, the large size of FPG could also pose an issue, as some elements of the model may not align with realities with a simpler organization, necessitating adaptations. Similarly, even in hospitals of comparable size, some rearrangements of the model might be needed to adapt the model to other data frameworks (e.g., other proprietary EHR software), especially for data extraction. On the same line, the use of ICD9-CM codes will facilitate the model’s diffusion in Italy, but it represents an obstacle to international exportation, where more updated and/or country-specific versions of ICD codes might be employed.

The protocol is also limited by its monocentric design, which might hinder the generalizability of findings across the country, and by the involvement of a selected number of departments that were opportunistically chosen, which might further compromise the generalizability of results.

Similarly, the inclusion of both inpatient and outpatient patients will ensure an equal representation of different care settings and of different care needs in the project, increasing the overall generalizability of results. At the moment, privacy laws in Italy, particularly concerning the processing of certain categories of data (such as those related to HIV positivity), present challenges for proactive initiatives like CareVax, leaving some very frail patients potentially excluded from their benefits. However, the CareVax system will be sufficiently flexible to include this kind of data, should these restrictive laws be reassessed in the future.

A valid criticism of CareVax might be that, despite its claim of offering an automated solution, it still necessitates a substantial amount of human effort. However, as healthcare digitization advances, the potential for easier patient contact and vaccination appointment scheduling becomes apparent, contributing to the widespread dissemination of personalized vaccine offers. The creation of a national vaccine registry, currently in progress, will make it easier to access information on patients’ vaccination status, allowing this model to be extended to patients without a family doctor in their region of residence.

In conclusion, this study contributes a novel approach to increasing vaccination coverage, offering a potential paradigm shift in public health practices. Despite its limitations, CareVax holds promise for broader application, emphasizing the need for ongoing refinement and adaptation to various healthcare settings.
