# Supplementary material for: Digital integration between hospitals and local health authorities for enhanced vaccination coverage among frail patients: the CareVax study protocol
Source: Front Public Health. 2025 Jan 30;13:1490244. doi: 10.3389/fpubh.2025.1490244 (PMC11822475; doi:10.3389/fpubh.2025.1490244)
Supplement: Supplementary file 1 [file Supplementary_file_1.docx]

**Carevax Questionnaire**

**Date** ...../...../...............

***Vaccine (if given multiple vaccinations in the same session mark each one)***

- COVID19 Vaccine
- Influenza Vaccine
- Pneumococcal Vaccine
- Herpes Zoster Vaccine
- Hepatitis B Vaccine

1) **Citizenship**: **❑** Italian ❑ Other (please, specify………………………………………)

2**)Residency ZIP Code** |__|__||__|__||__|

3) **Age**: |__|__|

4) **Sex:**

- - Woman
  - Man
  - Non Binary
  - Prefer not to say

5) **Marital Status**

- - Not Married or Divorced or Widowed
  - Married or in a Long-Term Relationship

6) **Highest level of formal education**

- None
- Primary School
- 1th grade Secondary School
- 2th grade Secondary School
- Bachelor degree or higher
- Prefers not to say

7) **For your health condition, what is the main clinic/department you are treated from at Fondazione Policlinico Gemelli (e.g. Nephrology, Gastroenterology)?**

..........................................................................................................................................................................................................................................................................................................................................................

8) **Last year, where you vaccinated against influenza?**

- Yes
- No

9) **For how long have you been living with your most severe disease?**

- Less than 2 years
- Between 2 and 5 years
- Between 5 and 10 years
- More than 10 years

10) **What is the gross annual income of your family?** (estimated)

- Less than 20 000 euro
- Between 20 001 and 50 000 euro
- Between 50 001 and 100 000 euro
- Between 100 001 and 150 000 euro
- More than 150 000 euro
- Prefers not to say

11) **Do you work or study in the healthcare sector?** (e.g. Doctor, Nurse, Pharmacist, etc )

- Yes
- No

1. **The following statements refer to your opinion on the vaccination pathway offered at the Fondazione Policlinico Gemelli and its organization from when the study was proposed to you until now. We ask you to indicate, for each of them, the degree of agreement/disagreement with them from 1 (Strongly disagree) to 5 (Strongly agree)**

|  | **1** | **2** | **3** | **4** | **5** |
| --- | --- | --- | --- | --- | --- |
| 1. Having my health information processed by a computerized system in order to assess my eligibility for vaccination, makes me fear for my privacy |  |  |  |  |  |
| 2. I am confident that all necessary steps have been taken in the vaccination pathway to protect my medical and health information |  |  |  |  |  |
| 3. I am confident in the ability of the medical staff to maintain privacy and confidentiality of my medical and health information |  |  |  |  |  |
| 4. The doctor treating me at the Fondazione Policlinico Gemelli was impactful in my decision to have this vaccination |  |  |  |  |  |
| 5. The vaccination appointment booking was simple, quick, and satisfactory |  |  |  |  |  |
| 1. 6. I received clear information about the place and time of vaccination |  |  |  |  |  |
| 1. 7. The waiting time for vaccination was acceptable |  |  |  |  |  |
| 1. 8. The vaccination area (vaccine administration box, waiting room, observation room) was comfortable |  |  |  |  |  |
| 1. 9. The vaccination area (vaccine administration box, waiting room, observation room) was comfortable |  |  |  |  |  |
| 1. 10. The vaccination area (vaccine administration box, waiting room, observation room) was adequate in protecting my privacy |  |  |  |  |  |
| 1. 11. I am generally satisfied with the organization of the vaccination pathway |  |  |  |  |  |

1. **The following statements refer to the health care personnel by whom you were assisted in the immunization pathway offered at the Policlinico Gemelli Foundation. Please indicate, for each of them, the degree of agreement/disagreement from 1 (Strongly disagree) to 5 (Strongly agree).**

|  | **1** | **2** | **3** | **4** | **5** |
| --- | --- | --- | --- | --- | --- |
| 1. 1. I received complete and clear information about the benefits and risks of vaccination from the medical staff , which enabled me to make an independent decision to vaccinate myself |  |  |  |  |  |
| 1. 2. I consider the medical staff of the vaccination clinic to be professional and competent |  |  |  |  |  |
| 1. 3. I consider the medical staff at the vaccine clinic polite and willing to listen |  |  |  |  |  |
| 1. 4. I consider the nursing staff of the vaccine clinic to be professional and competent |  |  |  |  |  |
| 1. 5. I consider the nursing staff at the vaccine clinic polite and willing to listen |  |  |  |  |  |

1. **The following statements refer to your opinions and attitudes about vaccination. Please indicate, for each of them, the degree of agreement/disagreement from 1 (strongly disagree) to 5 (strongly agree).**

|  | **1** | **2** | **3** | **4** | **5** |
| --- | --- | --- | --- | --- | --- |
| 1. Because of my pathology I could have become ill with the disease I was vaccinated against much more easily than others |  |  |  |  |  |
| 2. If I had become ill with the disease I was vaccinated against, the consequences for my health would have been severe |  |  |  |  |  |
| 3. If I had gotten sick with the disease I was vaccinated against, my chronic conditions would have gone through major complications |  |  |  |  |  |
| 4. I was undecided about vaccination because of doubts about the efficacy of the vaccine |  |  |  |  |  |
| 1. 5. I was undecided about this vaccination because of doubts about the safety of the vaccine |  |  |  |  |  |
| 6. I was undecided about this vaccination for fear of side effects |  |  |  |  |  |
| 7. I was undecided about this vaccination for fear of possible interactions with my therapy |  |  |  |  |  |
| 8. I felt embarrassed asking the doctor for clarification about the vaccine |  |  |  |  |  |
| 9. Before vaccination, I feared that the vaccine might cause the same diseases it tries to prevent |  |  |  |  |  |
| 10. If I had been offered to be vaccinated at a different center, I would have made the same choice |  |  |  |  |  |
| 11. I believe that if I had not participated in the Carevax study, I would still have taken action to carry out the vaccinations i had been given |  |  |  |  |  |

1. **We ask you to cross out your answers. You may choose more than one.**

What was influential in your choice to vaccinate?

- 1. 🞏 General Practitioner
  2. 🞏 Hospital Specialist treating me
  3. 🞏 Other physician
  4. 🞏 Official Institutional Communications in Tv/Radio
  5. 🞏 Television (newscasts, talk shows, etc.)
  6. 🞏 Institutional websites (Ministry, ISS, ASL, ...)
  7. 🞏 Non-institutional websites (online newspapers, blogs, forums, etc.).
  8. 🞏 Printed newspapers
  9. 🞏 Family/friends/acquaintances
  10. 🞏 Others(Example: Telegram, Facebook, Twitter, Instagram, etc.) Specify.....................................................................................................................................................................................................................................................................................
